# Thoracic Anaesthesia Practices in Turkey: A Survey Study

**DOI:** 10.5152/TJAR.2022.22042

**Published:** 2022-12-01

**Authors:** Bayram Doğan, Mediha Türktan, Ersel Güleç, Dilek Özcengiz

**Affiliations:** Department of Anaesthesiology, Çukurova University Faculty of Medicine, Adana, Turkey

**Keywords:** Anaesthesia, survey, thoracic surgery, Turkey

## Abstract

**Objective:**

In this survey study, we aimed to investigate thoracic anaesthesia practices in Turkey.

**Methods:**

The survey was sent to the members of the Turkish Society of Anesthesiology and Reanimation by e-mail. Participants were asked to answer 35 questions about their thoracic anaesthesia practice.

**Results:**

A total of 148 questionnaires were completed. Most of the participants preferred double-lumen endobronchial tube for one-lung ventilation. 69.6% of auscultation method and 45.9% of fiberoptic bronchoscope method were used to confirm the tube position. The most frequently used additional monitoring method was invasive blood pressure. Generally, intravenous anaesthetic agents were preferred for anaesthesia induction, and a combination of inhalation and intravenous agents was used for anaesthesia maintenance. Most of the participants used intraoperative lung-protective mechanical ventilation strategies. For postoperative analgesia, 75% of participants preferred regional analgesic techniques and 89.9% of them used routine opioid agents. In general, moderate amount of fluid was applied (57.4%), crystalloids were the first choice in fluid therapy, and intraoperative hypotension was generally treated with controlled intravenous fluid and vasoactive agents. The haemoglobin threshold value for blood transfusion was stated as 8 g dL^−1^ by 35.8% of participants.

**Conclusions:**

Our data showed that the anaesthesia management of thoracic surgery in Turkey is generally compatible with the current international guidelines. However, the following conclusion was reached: training on blood transfusion, the use of fiberoptic bronchoscope, regional techniques, and intraoperative additional monitoring would be beneficial, and a national consensus should be reached on the thoracic anaesthesia practice.

**Main Points**

- Choosing an anaesthetic agent, mechanical ventilation settings, calculating the fluid requirement, one-lung ventilation management, and providing effective analgesia are very important during thoracic surgery.
- Anaesthesia management for thoracic surgery in Turkey is generally compatible with the current international guidelines.
- Training on intraoperative monitoring, blood transfusion, the use of fiberoptic bronchoscopy, and regional techniques should be continued.
- Also, a national consensus needs to be established in terms of thoracic anaesthesia practice.

## Introduction

A detailed anaesthesia evaluation should be performed in the patients undergoing thoracic surgery. It is very important to choose an anaesthetic agent, mechanical ventilation (MV) settings, calculate the fluid requirement, one-lung ventilation (OLV) management, and provide effective analgesia. The goals that should be achieved during thoracic anaesthesiology are preoperative risk determination, provision of optimal preoperative conditions, prevention and treatment of hypoxia, and finally, planning the most appropriate anaesthesia and analgesia methods.

Practices in thoracic anaesthesia vary depending on the clinic and the preferences of the surgeon and the anaesthesiologists. The purpose of this study is to investigate thoracic anaesthesia practices in Turkey.

## Methods

This questionnaire-based study was sent to the members of the Turkish Society of Anesthesiology and Reanimation after approval by the Çukurova University Faculty of Medicine ethics committee. The survey was conducted via commercially available survey software (ww.survey.com) and sent to all members via e-mail to obtain an overview of thoracic anaesthesia clinical practices in Turkey. Respondents completed the questionnaire during the months of January and February 2021.

The target population was anaesthesiologists who had thoracic anaesthesia experience and actively working. After all participants were informed about the purpose of the survey, they were asked to answer 35 questions about their thoracic anaesthesia practices via the shared link.

### Statistical Analysis

Statistical analysis was performed using the Statistical Package for the Social Sciences software version 20.0 (IBM Corp, Armonk, NY, USA). Data were analysed using frequency distribution, chi-square, or Fisher exact tests. Categorical measures were summarised as numbers and percentages.

## Results

A total of 148 anaesthesiology physicians participated in our survey study. The type of hospital and duration of thoracic anaesthesia experience are shown in Table 1.

Most respondents (97.3%) reported a double-lumen tube (DLT) as the first-choice lung separation technique, and others were single-lumen endotracheal tube (2%) and EZ-blocker endobronchial blocker (0.7%). To verify the correct positioning of a lung separation device, 69.6% of participants used the auscultation method, 45.9% used fiberoptic bronchoscopy (FOB), and 8.8% used surgical observation. The majority of the participants (63.5%) preferred a DLT according to the surgical side (right DLT for all left-sided surgery, left DLT for all right-sided surgery). However, 33.1% of participants indicated the sole use of left DLTs, and 3.4% of the participants indicated the sole use of right DLTs.

Invasive arterial blood pressure monitoring was stated as mandatory by 85.8% of participants. The rate of routine central venous catheter placement was 27.7%. The other most common additional monitoring methods were temperature monitoring (23.6%), cerebral monitoring (6.1%), neuromuscular monitoring (4.1%), and dynamic cardiac output monitoring (1.4%).

Routine premedication was administered by 45.9% of participants. For anaesthetic induction, intravenous (iv) anaesthetics were most commonly preferred (98.6%). Forty-seven participants (31.8%) only maintained anaesthesia with an inhalation agent, 18 participants (12.2%) reported the sole use of iv anaesthesia, and 83 participants (56.1%) preferred the combination of inhalation and iv agents.

During both 2-lung ventilation (TLV) and OLV, tidal volume was adjusted according to ideal body weight by the majority of participants (82.4%). Mechanical ventilation settings during TLV and OLV are shown in Tables 2 and  3, respectively.

Of all the respondents, 75% preferred regional techniques for postoperative analgesia. The most preferred regional analgesia technique for post-thoracotomy pain was thoracal epidural analgesia (TEA). Most anaesthesiologists preferred iv analgesia in video-assisted thoracoscopic surgery (VATS) over regional techniques. Preferred postoperative analgesia methods for thoracotomy and VATS are shown in Table 4. The epidural catheter was often inserted before anaesthesia induction (85.9%), however, paravertebral block (PVB) and erector spinae plane block (ESPB) were applied by the anaesthesiologist before and after anaesthesia induction at a rate of 41.6% and 55%, respectively and by the surgeon during surgery at a rate of 3.4%. The routine systemic opioid usage rate was 89.9% and routine non-steroid anti-inflammatory drugs usage rate was 66.2%.

During OLV, most of the participants preferred a moderate amount of fluid administration (5 mL kg^−1^ h^−1^) and crystalloids were the first choice (Table 5). To manage hypotension, 57.4% of participants used controlled fluid administration and 40.6% of them preferred vasoactive agents. The most commonly used vasoactive agents were norepinephrine (54.1%) and ephedrine (35%).

The haemoglobin threshold value for initiating blood transfusion was specified as 8 g dL^−1^ by 35.8% of participants. This threshold value was stated as 7, 9, and 10 g dL^−1^ at the rate of 23%, 23.6%, and 17.6%, respectively.

The vast majority (83.1%) of the participants thought that there was a need for national consensus on thoracic anaesthesia practices.

## Discussion

Our survey study demonstrated that the most preferred lung isolation device was DLT, auscultation was still the first choice for confirmation tube placement, lung-protective ventilation strategies were frequently used, the moderate amount of fluid administration was the most preferred fluid strategy, regional techniques for postoperative analgesia are often used but iv analgesia used more than world standards for thoracic surgery in Turkey.

One of the still debated questions in thoracic anaesthesia is which is the best method to use to perform OLV. Narayanaswamy et al^1^ demonstrated that the collapse time of the lung was significantly shorter with left DLT compared to bronchial blockers (BB). Also, the number of repositions was lower in the left DLT group than in the BB group, but no difference was found in terms of lung collapse performance and quality. A DLT can be placed more easily than BB in a shorter time, and it provides the opportunity to aspirate the lungs separately but causes a higher rate of airway injury, sore throat, and hoarseness. Bronchial blockers are recommended in patients with difficult airway management, rapid sequential induction, and anticipated postoperative ventilator support.^2^ According to current survey studies, DLTs are still the first choice for lung isolation.^3-6^ In our study, DLTs were the most commonly used lung isolation method, but unlike in other studies, none of the participants preferred BB (except EZ BB). The reason why BBs are not preferred may be their high costs and FOB requirement during placements.

The location of DLT can be confirmed by chest movements and auscultation, but these clinical assessments do not always allow the detection of tube malposition. More than 30% of blindly placed DLTs are misplaced and FOB is the gold standard for verification of DLT placement.^7,8^ However, FOB is an expensive, time-consuming, and educational procedure, and ultrasonography (USG) may be a good alternative to FOB.^9^ Recent survey studies also demonstrated that FOB was the most commonly used verification method.^3,6^ In our study, none of the participants used USG to confirm the DLT placement, and the most commonly preferred verification methods were auscultation and FOB. However, the use of FOB was lower than the current literature recommendations. It was thought that this inadequacy might be due to financial policies, institution facilities, lack of experience, or the willingness of the surgical team to start surgery without wasting time.

The use of left or right DLT depends on the preference of the anaesthesiologist and surgeon. The high risk of collapse and obstruction due to the anatomical structure of the right upper lobe bronchus causes a decrease in the safety margin of the right DLTs. However, some studies have found that right and left DLTs have similar complication rates associated with malpositioning.^10^ The current survey studies demonstrated that right or left DLT depending on the surgery side was generally preferred.^3,11^ Consistent with the literature, the majority of our participants preferred right DLT for all left-sided surgery and left DLT for all right-sided surgery.

Thoracic surgery requires close intraoperative monitoring including blood pressure, oxygen saturation, and electrocardiography. The most commonly used additional monitoring methods are invasive arterial monitoring, central venous catheterization, temperature monitoring, neuromuscular monitoring, and transesophageal doppler.^3,5,11^ In our study, invasive blood pressure monitoring was frequently performed, but the other additional monitoring methods, especially temperature monitoring, without additional cost were used less frequently compared to the current literature.

Thoracic surgery patients may be elderly and have impaired respiratory function. Benzodiazepines may lead to excessive sedation, upper airway obstruction, prolongation of extubation time, decreased postoperative cognitive function, and delirium in this patient group. However, small doses of short-acting narcotics can be used during the application of regional blocks or in patients with anxiety.^12^ In our study, 45.9% of our participants applied routine premedication.

Airway reactivity of thoracic surgery patients may be higher than the other patient groups, and DLT placement, surgical airway manipulation, or instrumentation contribute to bronchoconstriction. It is very important to provide adequate anaesthesia depth during surgery. Modern inhalation agents minimally inhibit hypoxic pulmonary vasoconstriction, but there is no clinically significant difference in oxygenation between modern inhalation agents (MAC<1) and total iv anaesthesia (TIVA).^13^ Inflammatory markers in ventilated lungs were reduced with desflurane or sevoflurane compared to TIVA with propofol.^14^ However, no difference was demonstrated between inhalation and iv anaesthetic agents in terms of complications after thoracic surgery.^15,16^ Liu et al^11^ stated that the combination of inhalation and iv agents was more preferred for the maintenance of anaesthesia during thoracic surgery. In our study, while iv anaesthetics were the most commonly used induction agent (98.6%), the combination of inhalation and iv anaesthetics was frequently preferred for anaesthesia maintenance (56.1%).

Recent data suggest that prophylactic lung-protective mechanical ventilation strategies including lower tidal volume (6-8 mL kg^−1^ according to ideal body weight), adequate PEEP, and recruitment manoeuvers are associated with improved functional and physiological postoperative clinical outcomes.^17-19^ Female gender and obese patients are more exposed to non-protective ventilation practices with higher tidal volume calculated according to actual body weight. Although the appropriate PEEP level is still not known, zero PEEP is associated with atelectasis, pulmonary infection, and lung injury. Blank et al^20^ demonstrated that low TV without PEEP does not provide any benefit, but the combination of low TV and adequate PEEP improved postoperative outcomes after thoracic surgery. High oxygen concentrations increase free oxygen radicals, lead to absorption atelectasis, and increase postoperative pulmonary complications.^19^ The majority of our participants applied lung-protective ventilation strategies according to current literature information. During OLV, continuous positive airway pressure (CPAP) ≤5 cmH_2_O to the non-ventilated lung prevents atelectasis and reduces pro-inflammatory cytokine, but it is difficult to use in daily practice due to worsening surgical vision.^21,22^ In our study, the majority of the participants (83.8%) did not apply routine CPAP to the non-ventilated lung.

Mechanical ventilation with lower TV may also increase dead space and lead to hypercapnia. To prevent hypercapnia, respiratory rate is increased, but it may be harmful due to auto-PEEP, shortening of inspiratory : expiratory time, and increasing respiratory pressure.^18^ Lang et al^23^ demonstrated that hypercapnia decreased cytokine response, and therefore, moderate hypercapnia may be tolerated. According to lung-protective strategies, maximum of 70 mmHg PaCO_2_ levels is tolerated by healthy people, but it should be avoided in patients with pulmonary arterial hypertension, right heart dysfunction, or intracranial hypertension.^18,22^ In our study, the majority of the participants permitted maximum PaCO_2_ level was 45-54 mmHg. 

Pressure-controlled ventilation (PCV) increases arterial oxygenation by reducing the heterogeneity of ventilation and is an alternative mode to volume-controlled ventilation (VCV). Pressure-controlled volume-guaranteed ventilation (PCV-VG) combines the advantages of VCV and PCV and thus allows to avoid hypoventilation, as well as barotrauma and volutrauma.^24^ However, their superiority to each other in terms of postoperative pulmonary complications could not be demonstrated.^11^ Peak airway pressure higher than 35 cmH_2_O and plateau pressure higher than 25 cmH_2_O are considered harmful.^18^ Especially in patients with high airway pressure, despite correct tube placement, pressure-controlled ventilation modes may be preferred during OLV.^18^ In our study, the majority of participants preferred pressure-adjusted ventilation modes during OLV.

Excessive fluid administration is one of the most important risk factors after thorax surgery. In recent years, concerns about the volume-limiting fluid regimen include impaired tissue perfusion, organ dysfunction, acute kidney injury, and hypovolemia.^12^ A restrictive fluid regime may lead to perioperative oliguria, but it has not been confirmed to cause a risk of postoperative acute kidney injury.^25^ According to thoracic surgery Enhanced Recovery After Surgery (ERAS) protocol, very restrictive or liberal fluid regimes should be avoided and balanced crystalloids are the first choice.^12^ Thoracic surgery ERAS guidelines also suggest that intraoperative hypoperfusion can be prevented with the use of vasopressors and a limited amount of fluid.^12^ In our study, our participants frequently preferred a moderate amount of crystalloid administration to maintain euvolemia and controlled iv fluids or vasoactive agents in the management of intraoperative hypotension. Today, it is recommended to maintain Hb >8 g dL^−1^ for thoracic surgery. In our study, although 35.8% of participants determined the transfusion threshold value as 8 g dL^−1^, we think that this rate is not sufficient.

Although TEA is the gold standard for post-thoracotomy pain, new regional techniques such as PVB or ESPB are alternative methods due to less risk of complication. In our study, TEA was the first option (42.6%) for post-thoracotomy pain, but this rate was lower than the current literature. Other regional techniques were used more frequently, and the use of only iv analgesia was higher compared to existing studies.

Finally, 83.1% of our participants stated that a national consensus is needed on thoracic anaesthesia practices.

We accept that this survey study has some limitations. First, although the questionnaire was sent to all members and reminded twice, the response was low. Nevertheless, the number of participants is similar to the current survey studies. Second, the number of participants who have experience of below 10 years of thoracic surgery is higher than in the other studies. Experienced physicians did not volunteer to participate in our survey. Third, the questionnaire was limited to intraoperative management, and some components including antibiotic use, postoperative care, and thromboprophylaxis were not evaluated.

## Conclusion

This study is the first survey study in Turkey that contains detailed data on this specific subject. Our data showed that the anaesthesia management of thoracic surgery in Turkey is generally compatible with the current international guidelines and literature followed worldwide. However, it has been concluded that training on intraoperative monitoring, blood transfusion, the use of FOB, and regional techniques should be continued. Also, a national consensus needs to be established in terms of thoracic anaesthesia practice. We think that this survey study is important in terms of detecting our deficiencies in thoracic anaesthesia management and raising awareness of this issue in our country.

## Figures and Tables

**Table 1.** The Type of Hospitals and Number of Years of Thoracic Anaesthesia Experience

	Number of Participants (n)	%
Institutions		
State university	66	44.6
Training and research hospital	34	23
Public hospital	34	23
Private hospital	8	5.4
Foundation university	5	3.4
Private university	1	0.7
Thoracic anaesthesia experience (year)		
Less than 5 years	64	43.2
5-10 years	45	30.4
10-15 years	15	10.1
Over 15 years	24	16.2

**Table 2.** Mechanical Ventilation Settings During 2-Lung Ventilation

	Number of Participants (n)	%
Mechanical ventilation mode		
VCV	74	50.0
PCV	23	15.5
PCV-VG	51	34.5
Tidal volume		
8-10 mL kg^−1^	15	10.1
6-8 mL kg^−1^	112	75.7
4-6 mL kg^−1^	21	14.2
PEEP		
0	2	1.4
1-4 cmH_2_O	42	28.4
5-10 cmH_2_O	104	70.3

VCV, volume-controlled ventilation; PCV, pressure-controlled ventilation; PCV-VG, pressure-controlled ventilation-volume guaranteed; PEEP, positive end-expiratory pressure.

**Table 3.** Mechanical Ventilation Settings During 1-Lung Ventilation

	Number of Participants (n)	%
Mechanical ventilation mode		
VCV	56	37.8
PCV	42	28.4
PCV-VG	50	33.8
Tidal volume		
8-10 mL kg^−1^	0	0
6-8 mL kg^−1^	19	12.8
4-6 mL kg^−1^	129	87.2
PEEP to ventilated lung		
0	15	10.1
1-4 cmH_2_O	59	39.9
5-10 cmH_2_O	73	49.3
>10 cmH_2_O	1	0.7
PEEP to non-ventilated lung		
0	124	83.8
1-3 cmH_2_O	10	6.8
4-5 cmH_2_O	11	7.4
>5 cmH_2_O	3	2.0
Routine recruitment maneuver		
Yes	95	64.2
No	53	35.8
FiO**_2_**		
100%	24	16.2
80%-99%	25	16.9
60%-79%	57	38.5
40%-59%	42	28.4
Lowest accepted oxygen saturation		
95%	8	5.4
90%	78	52.7
85%	48	32.4
80%	14	9.5
Permissible PaCO**_2_** level		
Do not use permissive hypercapnia	30	20.3
45-54	82	55.4
55-64	29	19.6
65-74	7	4.7

VCV, volume-controlled ventilation; PCV, pressure-controlled ventilation; PCV-VG, pressure-controlled ventilation-volume guaranteed; PEEP, positive end-expiratory pressure; FiO_2,_ fraction of inspired oxygen; PaCO_2,_ partial pressure of carbon dioxide.

**Table 4.** Postoperative Analgesia Methods for Thoracotomy and VATS

	Number of Participants (n)		%	
	Thoracotomy	VATS	Thoracotomy	VATS
Thoracal epidural analgesia	63	18	42.6	12.2
Lumbar epidural analgesia	7	0	4.7	0
Paravertebral block	25	23	16.9	15.5
Erector spinae plane block	41	35	27.7	23.6
Intercostal block	43	38	29.1	25.7
Intrathecal morphine	17	7	11.5	4.7
Intravenous analgesia	124	129	83.8	87.2

VATS, video-assisted thoracoscopic surgery.

**Table 5.** The Fluid Management During 1-Lung Ventilation

	Number of Participants (n)	%
First choice in iv fluid regimen		
Crystalloid	145	98
Colloid	3	2
Preferred amount of fluid		
Liberal (>10 mL kg^−1^ h^−1^)	7	4.7
Moderate (5 mL kg^−1^ h^−1^)	85	57.4
Restrictive (3 mL kg^−1^ h^−1^)	56	37.9
First choice for intraoperative hypotension management		
Massive iv fluid	3	2.0
Controlled iv fluid	85	57.4
Vasoactive agent	60	40.6
